# Assessment of the Physicochemical Qualities and Prevalence of *Escherichia coli* and Vibrios in the Final Effluents of Two Wastewater Treatment Plants in South Africa: Ecological and Public Health Implications

**DOI:** 10.3390/ijerph121013399

**Published:** 2015-10-23

**Authors:** Olayinka Osuolale, Anthony Okoh

**Affiliations:** 1 SAMRC Microbial Water Quality Monitoring Centre, University of Fort Hare, Alice 5700, South Africa; E-Mail: aokoh@ufh.ac.za; 2 Applied and Environmental Microbiology Research Group, Department of Biochemistry and Microbiology, University of Fort Hare, Alice 5700, South Africa

**Keywords:** final effluent, wastewater, *Vibrio*, *E. coli*, physicochemical, public health

## Abstract

The final effluents of two wastewater treatment plants (WWTPs) in the Eastern Cape Province of South Africa were evaluated for their physicochemical and microbiological qualities over a period of 12 months. The physicochemical parameters assessed ranged as follows both plants. The ranges of values for the physicochemical are: pH (3.9–8.6), total dissolved solids (86.50–336.3 mg/L), electrical conductivity (13.57–52.50 mS/m), temperature (13–28 °C), nitrate (0–21.73 mg/L), nitrite (0.01–0.60 mg/L), orthophosphate (1.29–20.57 mg/L), turbidity (4.02–43.20 NTU), free chlorine (0.05–7.18 mg/L), dissolve oxygen (3.91–9.60 mg/L), biochemical oxygen demand (0.1–9.0 mg/L) and chemical oxygen demand (4.67–211 mg/L). The microbiological assessment for both WWTPs revealed the presence of *E. coli* in counts ranging between 0 and 1.86 × 10^4^ CFU/100 mL and *Vibrio* counts ranging between 0 and 9.93 × 10^3^ CFU/100 mL. We conclude that these WWTPs are important point sources of pollution in surface water with potential public health and ecological risks.

## 1. Introduction

The removal of impurities present in wastewater in the form of suspended solids, organic substances, and nutrients and removal of pathogens are some of the basic purposes of wastewater treatment. The treatment of wastewater is important to prevent pollution of the environment and waterbodies. More important is to protect the public health by safeguarding against the spread of pathogenic diseases [1,2,3]. Municipal wastewater is a matrix consisting of raw sewage composed of micro-organisms, biodegradable organic materials and compounds, metals and other inorganic materials [4]. While routine wastewater treatment diminishes the load of enteric organisms and organic nutrients, limitations such as faulty machinery and lack of trained personnel faced during treatment processes can reduce removal efficiency such that wastewater effluents are still regarded as reservoirs of enteric pathogens [5]. If treatment fails, effluents of poor microbial quality enter public waters like rivers and dams, posing a risk to human and aquatic ecosystems [6]. 

The presence or abundance of faecal coliform in wastewater final effluents is a feeble index of the presence of human pathogens in wastewater entering environmental waters.”Regardless of this, utilization of faecal coliforms for water quality testing purposes is universal [7]. Certain clonal groups of *E. coli* with virulence qualities of uropathogenic strains were reported to survive the wastewater treatment processes [8,9]. Also, pathogenic strains of *Vibrio* have been reported to be isolated from effluents in WWTP in the Eastern Cape of South Africa and some other places [10,11,12,13]. Efficient removal of pathogens from wastewaters is needful to avert public health disasters due to contaminated water sources [14]. It has been reported that conventional municipal wastewater treatment without effective tertiary treatment such as filtration or disinfection may constitute a danger for public health from enteric pathogens, bacterial or otherwise [14]. Even so, some enteric bacteria have been found to better survive the activated sludge as well as the trickling filter treatment processes [15,16]. The inactivation rates of enteric bacteria by chlorine treatment have been found to be adequate where effluent treatment is efficient [17] while the absence of organic matter reduces the resistance of the bacteria in treated effluent during the disinfection process [18].

In South Africa, many communities still source their water from untreated surface water and ground-water for their daily water needs [19,20], which is frequently contaminated by wastewater effluents with faecal pollution [4]. Exposure to contaminated water, either by consumption or contact activities, may lead to grievous significant health risk to humans with myriad records of disease outbreaks and poisonings [21]. In this paper, we report on the physicochemical qualities and prevalence of *E. coli* and *Vibrio* species in the final effluents of two wastewater treatment plants in the Eastern Cape Province of South Africa and hypothesize that the two wastewater treatment plants effluents are treated to the acceptable standard as recommended by the Department of Water Affairs, South Africa.

## 2. Experimental Section 

### 2.1. Study Area and Sampling Procedure 

The two study plants are located in the Eastern Cape Province of South Africa. WWTP-A uses the activated sludge system with treatment design capacity of 5 ML/day that is located at a geographical location of longitude 33° 00′ 59″S and latitude 27° 51′ 48″E and (DWAF, 2009), while WWTP-B is located at the geographical coordinates of longitude 27° 23′ 47″ S and latitude 32° 85′ 36″ E, uses the trickling filter system and has a design capacity of about 8 ML/day.

Samples were collected on a monthly basis from the treated final effluent (FE) for a period of 12 months (September 2012 to August 2013). Samples were collected in sterile 1.7 L Nalgene bottles into which 1 mL of 10% sodium thiosulphate had been added to de-chlorinate the sample. Samples were then transported to the University of Fort Hare, Alice, South Africa, in chiller boxes containing ice to the Applied and Environmental Microbiology Research Group (AEMREG) laboratory for analysis within 6 h of collection. Some physicochemical parameters like temperature, pH, electrical conductivity (EC), total dissolve solid (TDS), and dissolved oxygen (DO), were all determined *in situ* using the multi-parameter ion specific meter (Hanna-BDH laboratory supplies). A microprocessor turbidity meter (HACH Company, model 2100P) was used to determine *in situ* the turbidity. The standard photometric methods were determined in the laboratory to measure the concentrations of orthophosphate, nitrate, nitrite, and chemical oxygen demand (COD) using the Spectroquant Pharo 100 Photometer (Merck Pty. Ltd, South Africa). Prior to analysis, samples for COD analysis were digested with a thermoreactor (Model TR 300, Merck Pty. Ltd., South Africa). Biochemical Oxygen Demand (BOD) was measured using a BOD meter (Hach 2100P) following the BOD_5_ procedure [22]. As a quality control step, the instruments were calibrated following the manufacturer’s instructions. The nutrients, including phosphate, nitrate and nitrite were analyzed in the laboratory using standard methods as described by the supplier (Merck, VWR International, Poole, UK).

Bacteriological analysis of the effluent samples was done by membrane filtration according to the method of SANS [23]. For *E. coli* enumeration, filters were placed on *E. coli* coliforms chromogenic agar and incubated at 37 °C for 24 h. and reported as CFU/100 mL [23]. Thiosulfate Citrate Bile Salts Sucrose agar (TCBS) was used for the enumeration of presumptive *Vibrio* species. All analyses were done in triplicates. 

### 2.2. Statistical Analysis

The One-sample T test was used to test the level of significance between the determined parameters and the DWAF effluent standards. The statistical significance was set to *p* < 0.05.

## 3. Results

Results for the evaluated physicochemical parameters of effluents of the two wastewater treatments plants are compared against the South African recommended standards for effluent discharge Table 1. The results of the physicochemical qualities are shown for WWTP-A in Table 2. The pH of the final effluent samples ranged between 7.4 and 8.6. At the WWTP-B, the pH of the final effluent samples ranged between 3.9 and 7.5. Both pH levels from the two WWTPs were statistically significant at *p* < 0.05 (*p* = 0.00). The pH level remained stable within the recommended pH limit throughout the period of this study. Dissolved oxygen concentration varied between 3.9 mg/L and 9.6 mg/L. It was relatively low for most of the months sampled at WWTP-A, while dissolved oxygen concentration at WWTP-B varied between 6.9 mg/L and 9.4 mg/L. The level of significance for DO could not be tested for because currently there is no set standard for it in DWAF. The BOD of the effluents ranged from 3.1 mg/L to 9.0 mg/L. The measured BOD_5_ at WWTP-A was above the limit of 2 mg/L expected for the maximum amount of oxygen required to be utilized. The month of December had the highest BOD_5_ level observed as shown in Table 2. At WWTP-B (Table 3), the BOD of the effluents ranged from 0.1 mg/L to 7.4 mg/L. The BOD_5_ was <2 mg/L, which falls within the amount of oxygen expected to be utilized for the months of September 2012, February and May 2013 with the other months having high concentration of BOD_5_. Chemical oxygen demand values ranged from 4.67 mg/L to 211 mg/L at the WWTP-A. The plant had 33% of the samples analyzed exceeding the recommended limit while the rest of the samples were slightly higher in COD concentration levels. At the 95% confidence interval, the COD level was insignificant (*p* = 0.81). The highest COD concentrations were observed in the months of May all through to August. COD values at WWTP-B ranged from 10.33 mg/L to 88.33 mg/L and 83% of samples analyzed were within the recommended limit with the months of September 2012 and April 2013 having the highest COD level. At 95% confidence interval, the COD level was significant (*p* = 0.00). The TDS value ranged between 253 mg/L and 336.3 mg/L at WWTP-A while at WWTP-B, the TDS value ranged between 86.8 mg/L and 127.5 mg/L. The TDS levels from the two WWTPs were statistically significant at *p* < 0.05 (*p* = 0.00). At WWTP-A, nitrite levels ranged between 0.19 mg/L and 0.60 mg/L and nitrate level ranged between 0.00 mg/L and 6.13 mg/L. The measured values for both nitrite and nitrate were in compliance with the permissible general limits of 15 mg/L and 1.5 mg/L values respectively for effluent discharge. The nitrite and nitrate levels at WWTP-A were statistically significant at *p* < 0.05 (*p* = 0.00). Nitrite levels ranged between 0.01 mg/L and 0.43 mg/L and were within permissible limit at WWTP-B. The nitrate level ranged between 8.03 mg/L to 18.70 mg/L at the WWTP-B. In few instances was the nitrate level above the 15 mg/L set limit as observed in the final effluent for the months of September, November 2012, and March and April 2013. The nitrite levels at this plant was statistically significant at *p* < 0.05 (*p* = 0.00) while the nitrate level was insignificant at *p* = 0.21. The recommended permissible level of orthophosphate is 10 mg/L. The phosphate level ranged from 3.89 mg/L to 20.57 mg/L for WWTP-A, while it ranged between 1.29 mg/L to 11.37 mg/L at the WWTP-B.

The phosphate level at WWTP-B was statistically significant at *p* < 0.05 (*p* = 0.00), but it was not for the WWTP-A plant (*p* = 0.35). The free chlorine for WWTP-A ranged between 0.05 mg/L and 0.71 mg/L, while it ranged between 0.06 mg/L to 7.18 mg/L at the WWPT-B. A general limit of 0.25 mg/L and special limit of 0 mg/L was set for free chlorine. The chlorine values at the two WWTPs were statistically insignificant (*p* > 0.05). About 67% of the WWTP-A samples had low free chlorine concentration below the recommended set limit while WWPT-B samples had acceptable levels of free chlorine concentration. There were instances where high chlorine overdosing was recorded at both sites. The turbidity for WWTP-A ranged between 4.76 NTU and 43.20 NTU, while it ranged between 4.02 NTU to 24.33 NTU at the WWTP-B. The DWAF regulation does not state any value to be used as quality guideline for turbidity for effluent except only for domestic use where a set value of 0–1 NTU was specified, 3 NTU was specified for recreational water and 25 NTU for aquaculture. About 83.3% and 41.7% of the samples analyzed exceeded the set limit for both WWTP-A and WWTP-B respectively. Electrical conductivity ranged from 39.43 mS/m to 52.50 mS/m for WWTP-A. It was 13.57 mS/m to 19.90 mS/m at the WWTP-B. The EC values were statistically significant for both WWTPs for the regulatory set limits (general limits and special limits) at *p* < 0.05 (*p* = 0.00).

**Table 1:** List of recommended parameters and limits.

Parameters, Units	Regulatory Guidelines		
	General Limit	Special Limit	Reference
Total Dissolved Solid (mg/L)	450		[24]
pH	5.5–9.5	5.5–7.5	[25]
Electrical Conductivity (mS/m)	70 above intake to a maximum of 150	50 above background Receiving water, to a maximum of 100	
Temperature ( °C)	natural ambient water temperature of the receiving water resource should not increase by more than 2–3 degrees Celsius		
Free chlorine (mg/L)	0.25	0	
Nitrite (NO_2_^−^) (mg/L)	15	1.5	
Nitrate (NO_3_^−^) (mg/L)	15	1.5	
Phosphate (P) (mg/L)	10	1	
Chemical Oxygen Demand (COD) (mg/L)	75 after removal of algae	30	
Biochemical Oxygen Demand (BOD)	3–6		[26]
Dissolved Oxygen (mg/L)	≥5		[26]
Turbidity (NTU)	<5 NTU		[27]

The counts of presumptive *E. coli* and *Vibrio* are as shown in Table 4 for both plants. The presumptive *Vibrio* counts ranged between 0 and 9.93 × 10^3^ CFU/100 mL and were detected in 75% of the effluent samples analyzed. Presumptive *E. coli* counts ranged between 0 and 1.85 × 10^5^ CFU/100 mL with the month of June 2013 having the highest counts, and it was detected in 83.3% of the effluent samples analyzed, with 58.3% of the samples exceeding the general limit of 1000 CFU/100 mL as recommended by DWAF [25]. In the treatment WWTP-B, the presumptive *Vibrio* counts ranged between 0 and 1.44 × 10^3^ CFU/100 mL and *Vibrio* was detected in 91.7% of the effluent samples analyzed with all the samples below the 1000 CFU/100mL limit.. The highest *Vibrio* count was in the month of August 2013 while there was no count in the month of May 2013. Presumptive *E. coli* counts ranged between 0 and 1.86 × 10^4^ CFU/100 mL with the highest counts in August 2013. *E. coli* was detected in 75% of the effluent samples analyzed, with 91.7% of the samples below the general limit of 1000 CFU/100 mL. The *Vibrio* counts were statistically significant for both WWTPs for the regulatory limits set for feacal coliform at 1000 CFU/100 mL at *p*<0.05 (*p* = 0.00) while the *E. coli* counts were not significant at the 1000 CFU/100mL for both WWTPs (*p* = 0.17; *p* = 0.67).

**Table 2:** The average means of the measured physiochemical parameters for the WWTP-A.

Parameters	September 2012	October 2012	November 2012	December 2012	January 2013	February 2013	March 2013	April 2013	May 2013	June 2013	July 2013	August 2013
**pH**	7.4 ± 0.2	7.4± 0.1	8.1 ± 0.1	8.6 ± 0.2	7.4± 0.1	8.3 ± 0.2	8.2 ± 0	8.4 ± 0.1	8.4 ± 0.1	8.3 ± 0.2	7.4 ± 0.1	7.6 ± 0
**Turbidity (NTU)**	16.7 ± 0.60	16.7 ± 0.8	7.6 ± 0.4	11.1 ± 0.3	5.4 ± 0.2	11.9 ± 0.1	13 ± 1.2	4.8 ± 0.1	9.9 ± 0.1	43 ± 0.3	42.3 ± 3.8	26.3 ± 0.6
**EC (µS/cm)**	525 ± 4	397 ± 2	454 ± 1	404.7 ± 4	419.7 ± 4.9	428.3 ± 5.9	439 ± 5.6	437 ± 4.7	464 ± 33.7	442 ± 16.1	394.3 ± 3.8	505.3 ± 3.2
**TDS (mg/L)**	336 ± 2	254 ± 1	291 ± 1	259 ± 2.6	268.3 ± 3.8	274.3 ± 4.2	282 ± 2.5	279 ± 3.2	298 ± 21.8	282 ± 10.4	253 ± 2.6	323.7 ± 2.1
**DO (mg/L)**	5.5 ± 0.1	6.2 ± 0.40	5.0 ± 0.2	9.6 ± 0.3	4.4 ± 0.1	4.8 ± 0.3	4.7 ± 0.1	5.0 ± 0.1	4.5 ± 0.1	4.0 ± 0.4	3.9 ± 0.1	5.0 ± 0.1
**Temp. (°C)**	24 ± 2	19 ± 1	23 ± 0	22 ± 0.2	28 ± 0.4	21 ± 0.4	25 ± 0.4	24 ± 0.6	24 ± 0.6	19 ± 0.3	19 ± 0.6	17 ± 0.7
**Free chlorine (mg/L)**	0.71 ± 0.1	0.13 ± 0.10	0.05 ± 0	0.05 ± 0	0.17 ± 0	0.24 ± 0	0.25 ± 0	0.13 ± 0	0.1 ± 0	0.05 ± 0	0.14 ± 0.1	0.05 ± 0
**BOD (mg/L)**	4.36 ± 0.26	4.95 ± 0.40	4 ± 0.1	8.99 ± 0.5	3.4 ± 0.2	3.76 ± 0.3	3.93 ± 0.1	4.0 ± 0.2	3.23 ± 0.2	3.08 ± 0.8	3.42 ± 0.1	4.35 ± 0.6
**Nitrite (mg/L)**	0.77 ± 0.02	0.60 ± 0.1	0.46 ± 0	0.4 ± 0	0.25 ± 0	0.2 ± 0	0.23 ± 0	0.37 ± 0	0.2 ± 0	0.22 ± 0	0.2 ± 0	0.21 ± 0
**Nitrate (mg/L)**	12.50 ± 4.39	4.47 ± 0.40	5.1 ± 0.4	5.17 ± 1	4.6 ± 0.6	4.83 ± 0.5	6.13 ± 1.3	5.2 ± 1.4	4.8 ± 1.7	4.77 ± 0.5	0 ± 0	4 ± 0.1
**Phosphate (mg/L)**	1.56 ± 0.09	4.80 ± 0.10	4.4 ± 0	6.57 ± 0.3	5.35 ± 0	16.47 ± 1.9	16.77 ± 0.8	3.89 ± 0.1	4.29 ± 0.1	4.97 ± 0.1	4.21 ± 0.1	20.57 ± 0.5
**COD (mg/L)**	39 ± 8	45 ± 1	71 ± 5	42.3 ± 7	35.7 ± 7	61.8 ± 5	4.67 ± 5	59 ± 8.7	163.33 ± 135.1	173 ± 66.6	199 ± 75	211 ± 74.9

**Table 3:** The average means of the measured physiochemical parameters for the WWTP-B

Parameters	September 2012	October 2012	November 2012	December 2012	January 2013	February 2013	March 2013	April 2013	May 2013	June 2013	July 2013	August 2013
pH	3.9 ± 0.1	6.8 ± 0.1	7 ± 0	7.4 ± 0	6.8 ± 0.1	7.4 ± 0	6.9 ± 0	7.5 ± 0.2	4.8 ± 0.1	6.5 ± 0	5.7 ± 0.1	6.2 ± 0.1
TURBIDITY (NTU)	12.8 ± 1.50	5.8 ± 0.4	4.0 ± 0.4	12.8 ± 0.1	5.5 ± 0.5	5.5 ± 0.3	6.0 ± 1.7	5.6 ± 0.6	5.7 ± 1	17 ± 0.3	24.3 ± 0.6	21 ± 1.7
EC((µS/cm)	179 ± 2	155 ± 3	166 ± 1	161.8 ± 14.8	138.5 ± 3.1	135.7 ± 1	149 ± 3.8	147 ± 2	199 ± 4.6	159 ± 14.6	150.3 ± 5	173.7 ± 3.4
TDS (mg/L)	114 ± 2	99 ± 2	106 ± 0	103.8 ± 9.4	88.6 ± 2	86.8 ± 0.6	95 ± 2.5	94 ± 1.4	127 ± 3.1	102 ± 9.2	96.2 ± 3.3	111 ± 2.2
DO (mg/L)	7.9 ± 0.1	8 ± 0.1	7.9 ± 0	7.5 ± 0.1	7.5 ± 0.1	7.1 ± 0.1	6.9 ± 0.1	8.1 ± 0.1	8.4 ± 0.1	8.3 ± 0	9.3 ± 0	9.4 ± 0.2
TEMP.(°C)	20 ± 2	25 ± 4	25 ± 1	21 ± 0.2	22 ± 0.2	24 ± 2.3	25 ± 0.3	18 ± 0.3	16 ± 0.5	16 ± 0.6	13 ± 0.1	14 ± 0.8
FREE CHLORINE (mg/L)	2.31 ± 0.5	0.31 ± 0	0.36 ± 0	0.09 ± 0	0.22 ± 0	0.21 ± 0	0.16 ± 0	0.14 ± 0	7.18 ± 0.4	0.14 ± 0	0.06 ± 0	0.13 ± 0
BOD (mg/L)	0.13 ± 0.10	5.12 ± 1.80	4.48 ± 0.3	6.76 ± 0.1	4.96 ± 0.2	1.84 ± 2.1	4.9 ± 0.8	5.1 ± 1.7	0.37 ± 0.2	7.39 ± 0.4	6.65 ± 0.5	7.02 ± 0.3
NITRITE (mg/L)	0.18 ± 0	0.09 ± 0.10	0.01 ± 0	0.43 ± 0	0.16 ± 0	0.18 ± 0	0.04 ± 0	0.17 ± 0	0.18 ± 0	0.24 ± 0	0.18 ± 0	0.2 ± 0
NITRATE (mg/L)	17.87 ± 2.2	12.5 ± 0.4	16.5 ± 1.6	8.03 ± 1.6	10.87 ± 0.3	11.37 ± 0.7	18.7 ± 1.7	15.87 ± 1.9	13.97 ± 1.6	10.47 ± 1	14.53 ± 2.9	14.37 ± 2
PHOSPHATE (mg/L)	3.3 ± 0.1	2.29 ± 0.20	3.22 ± 0.1	2.66 ± 0.1	1.29 ± 0.1	1.52 ± 0.1	9.8 ± 0.3	11.37 ± 1.2	2.8 ± 0.1	3.19 ± 0	3.02 ± 0	2.79 ± 0
COD (mg/L)	82 ± 28	23 ± 1	11 ± 3	29.67 ± 15.3	27 ± 36.4	10.33 ± 5	54 ± 47.3	88.33 ± 9.5	47.33 ± 5	13 ± 17.3	22.33 ± 16.8	33.67 ± 13.3

**Table 4:** Mean bacterial counts for the wastewater treatment plants.

Sampling Site	Target Bacteria (CFU/100mL)	Sep-12	Oct-12	Nov-12	Dec-12	Jan-13	Feb-13	Mar-13	Apr-13	May-13	Jun-13	Jul-13	Aug-13
**WWTP- A**	*E. coli*	0	2.80 × 10^4^	1.30 × 10^2^	1.10 × 10^3^	3.33 × 10^2^	0	1.60 × 10^4^	2.10 × 10^2^	2.09 × 10^4^	1.85 × 10^5^	6.00 × 10^3^	1.29 × 10^4^
	*Vibrio* spp.	0	7.7 × 10^3^	4.6 × 10^3^	4.10 × 10^3^	6.00 × 10^3^	0	65	7.83 × 10^3^	4.30 × 10^3^	9.93 × 10^3^	8.40 × 10^3^	0
**WWTP-B**	*E. coli*	1	0	0	1.31× 10^3^	1.0	2	27	36	0	7	1.67 × 10^2^	1.86 × 10^4^
	*Vibrio* spp.	1	1	1.8 × 10^1^	7.00 × 10^2^	1	1	2	118	0	59	3.67 × 10^2^	1.44 × 10^3^

## 4. Discussion

One of the ways to evaluate water quality is to measure water quality variables and assess values for benchmarks, such as guidelines. The South African (SA) effluent discharge guidelines is outlined in National Water Act [25]. The outcome of the physicochemical analysis showed that six (6) out of the twelve (12) parameters tested for at WWTP-A complied with the South African (SA) effluent discharge standard limits as outlined in DWAF [25]. The parameters within the recommended limits were pH, TDS, nitrates, nitrites, orthophosphates and temperature. Other parameters like COD, EC and free chlorine did not meet the SA set limits for effluent discharge. Due to the lack of regulatory standards, dissolved oxygen (DO), BOD and turbidity were compared against other international physicochemical standards for discharged effluents as there are no set limits for these parameters in SA. These three (3) parameters were found not to comply with the EU directives on DO and BOD [28,29] or the WHO guidelines on turbidity [30]. It has been reported that each of the physicochemical parameters analyzed has synergetic effects on the others, which has some impact on water quality, as well as the use of the water [31,32,33,34,35]. The discharge effluents with a low dissolved oxygen, high BOD and turbidity signify potential danger to the receiving surface water. Low concentrations of dissolved oxygen, when combined with the presence of toxic substances, may lead to stress responses in aquatic ecosystems because the toxicity of certain elements is increased by low concentrations of dissolved oxygen [36]. Low DO content of treated effluent suggested an increase in the organic matter content [37]. The BOD is an important indication of organic pollution and the number of organisms increases in the breaking down of organic substances, so the demand for oxygen increases proportionately [37] and this can lead to an oxygen starvation of the body, thus killing the aquatic animals [38]. In addition, high COD also have similar effect to that of BOD [37]. At the WWTP Plant B, the nitrate, free chlorine and turbidity, as with the nine (9) other physicochemical factors, failed to comply with the SA regulatory limits and WHO guidelines. Elevated levels of nitrates can result in eutrophication, giving rise to increase algae growth and eventually leading to reduced dissolved oxygen levels in the water [39]. Turbidity of the final effluent can serve as a measure of treatment efficiency [40,41]. A treated turbid water indicates problems with treatment processes, particularly sedimentation [27]. For an efficient disinfection process to result in the removal of pathogenic bacteria and coliphages, low turbidity is required [42]. The effectiveness of chlorine decreases during disinfection in water with excessive turbidity. High turbidity and BOD causes increased chlorine demand; this may occur or be caused by the inadequate treatment of influent [43]. Osode [33] and Odjadjare *et al.* [44] also reported compliance of some of the WWTPs in the Eastern Cape Province with respect to these same physicochemical parameters like the pH, TDS, nitrate, nitrite, orthophosphate and temperature. Similarly, cases of non-compliance with regards to the non-compliant parameters reported above were found in some WWTP treatment plants in the Eastern Cape [45,46]. The non-compliance of some of these physicochemical parameters such as DO, BOD, COD and turbidity suggests that organic matter is present in high amounts in the effluent discharge. The continuous discharge of this poor quality effluent will contribute to the eutrophication of the surface water body and subsequently lead to the death of aquatic organisms. There is also the danger of cancerous substances present in the water, which form as a result of reaction of organic compounds with chlorine during the disinfection process [47,48]. 

The high counts of *E. coli* and *Vibrio* suggests the possible presence of potential pathogens in the final effluents, and in this regard the WWTP-A poses a serious threat to surface water and its environment (Table 4). The *E. coli* counts of 58% of the samples were over 1000 CFU/100 mL greater than the set guidelines, and these same effluents are meant to be discharged into the environment. The abundance of *E. coli* in the effluent presents a gloomy picture of the state of this wastewater treatment plant as the discharge of effluent from wastewater treatment plant(s) (WWTPs) could have major damaging effects on the health of aquatic ecosystems [49]. The *E. coli* counts at WWTP-B ranged between 0 and 1.86 × 10^4^ CFU/100 mL for the final effluent. About 83.3% of the analyzed samples complied with the effluent discharge limit using the faecal coliform set limit. Two of the months (December 2012 and August 2013) failed to meet the set limit. One of the months (December 2012) was as a result of inadequate treatment due to rain run-off into the treatment plant, which affected the treatment process. A possible cause of the high counts of *E. coli* observed in August 2013 was suspected to be the lack of disinfection during this month. Inadequate disinfection processes or a poorly operated treatment plant can result in multiplication or survival of various micro-organisms in already treated wastewater effluent, as they make their way into the environment [9]. Previous studies have reported the isolation of *E. coli* from treated effluent in the Eastern Cape [42]. U.S. EPA [50] recommends *E. coli* as the best indicator of health risk from water contact. Though they are considered harmless, they point to the likely presence of pathogens such as viruses and protozoans found living in human and animal digestive systems [50]. Hence, their presence in both WWTP final effluents suggest that pathogenic microorganisms might also be present and discharged in water bodies and the domestic use of such water might be a health risk.

The presumptive *Vibrio* species counts range from 0 to 1.4 × 10^3^ CFU/100 mL for the WWTP-B. The faecal coliforms guideline was used as the base limit for the evaluation of the *Vibrio* spp. Based on the calculated average for *Vibrio* spp., the WWTP-B complied with the general limit for the permissible amount of faecal coliform allowed for effluent to be discharged but failed for the special limit. Also, in all the samples analyzed for this plant, 91.7% of the Vibrio counts were less than the 1000 CFU/100 mL allowed for effluent discharged for the 11 months sampled. However, one of the months failed to comply with the 1000 CFU/100 mL set limit and the reason was as a result of no chlorine disinfection. The WWTP-A *Vibrio* counts range from 0 to 9.9 × 10^3^ CFU/100 mL with an average count of 3.8 × 10^3^ CFU/100 mL. Many of the samples (58.3%) analyzed failed to comply with the set limit. In this study, it was observed that the WWTP-A had a high prevalence of *Vibrio* in contrast to the WWTP-B, which had a very low prevalence of the organism in all the samples analyzed. In a recent work by Ye and Zhang [51] in Hong Kong, high prevalence of *Vibrio* was reported in the effluent of the studied treatment plant. The physicochemical parameter values measured at the WWTP-A are high, and this provides a suitable environment for the microbial community to proliferate in the treated effluent. Wani *et al.* [3] found that a treated effluent with relatively high physicochemical level provided an enabling environment for growth of faecal coliform. Rojas and Hazen [52] demonstrated that *Vibrio cholerae* could survive in effluent provided that the right optimum conditions suitable for growth exist. A similar study by Wennberg *et al.* [53] compared the survival of *Vibrio cholerae* and *Vibrio parahaemolyticus* in treated and untreated water and found that the bacteria proliferated in the treated water.

## 5. Conclusions

The peculiar challenges facing the water and sanitation sector in South Africa are still paramount; lack of access to clean water, health-related diseases associated with unsafe water and gross pollution to surface water highlight the need for the South Africa Department of Water Affairs (DWAF) to begin to review existing water policy and the enforcement needed to meet the standards set by other developed nations if they are to overcome these challenges. The year of millennium development goals (MDG) is 2015, and goal 7 stipulates ensuring environmental sustainability. The WWTP-A fared poorly in the treatment of its wastewater, and this result reveals the severe pollution resulting from organic matter in the final effluent, while the WWTP-B was efficient in treating its wastewater. The non-compliance of the former WWTP presents it as a plant that poses a potential danger to public health. Thus the outcome of this study definitely suggests an urgent need for pollutant reduction inputs into surface water and the need to upgrade existing wastewater treatment plants.
